# Integrated Cancer Screening Performance Indicators: A Systematic Review

**DOI:** 10.1371/journal.pone.0161187

**Published:** 2016-08-12

**Authors:** Silvina C. Mema, Huiming Yang, Marcus Vaska, Sherry Elnitsky, Zhichang Jiang

**Affiliations:** 1 Interior Health Authority, Kelowna, British Columbia, Canada; 2 Alberta Health Services, Calgary, Alberta, Canada; Duke University, UNITED STATES

## Abstract

Cancer screening guidelines recommend that women over 50 years regularly be screened for breast, cervical and colorectal cancers. Population-based screening programs use performance indicators to monitor uptake for each type of cancer screening, but integrated measures of adherence across multiple screenings are rarely reported. Integrated measures of adherence that combine the three cancers cannot be inferred from measures of screening uptake of each cancer alone; nevertheless, they can help discern the proportion of women who, having received one or two types of screening, may be more amenable to receiving one additional screen, compared to those who haven't had any screening and may experience barriers to access screening such as distance, language, and so on. The focus of our search was to identify indicators of participation in the three cancers, therefore our search strategy included synonyms of integrated screening, cervical, breast and colorectal cancer screening. Additionally, we limited our search to studies published between 2000 and 2015, written in English, and pertaining to females over 50 years of age. The following databases were searched: MEDLINE, EMBASE, EBM Reviews, PubMed, PubMed Central, CINAHL, and Nursing Reference Center, as well as grey literature resources. Of the 78 initially retrieved articles, only 7 reported summary measures of screening across the three cancers. Overall, adherence to cervical, breast and colorectal cancer screening ranged from around 8% to 43%. Our review confirms that reports of screening adherence across breast, cervical and colorectal cancers are rare. This is surprising, as integrated cancer screening measures can provide additional insight into the needs of the target population that can help craft strategies to improve adherence to all three screenings.

## Introduction

Screening has been defined as “the presumptive identification of unrecognized disease or defect by the application of tests, examinations or other procedures that can be applied rapidly.”[1] Screening for certain types of cancer can reduce death rates and health care costs. Cancer is set to become a major cause of morbidity and mortality in the coming decades in every region of the world; cancer screening coupled with other targeted interventions such as primary prevention of risk factors, vaccination, and effective treatment programs can ameliorate the projected cancer burden.[2]

Most major medical organizations [3] recommend a population approach to screening for cervical, breast and colorectal cancers for women over 50 years of age. Where available, organized screening programs set participation targets for each cancer screening, develop strategies to reach those targets and monitor the impact of the strategies with performance indicators of participation which ultimately reflect program acceptance and effectiveness.

**Fig 1:**
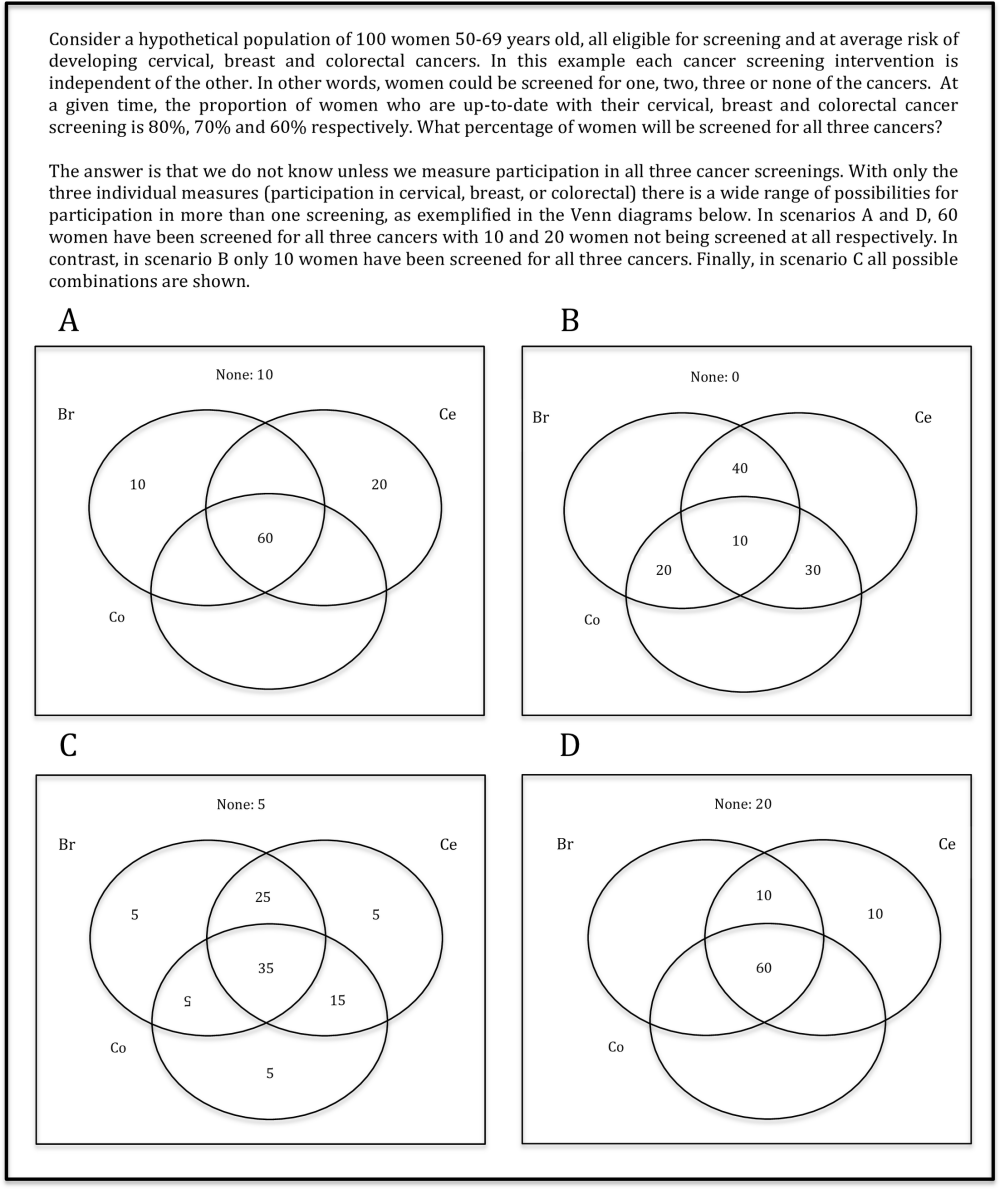
Hypothetical scenarios of cancer screening participation.

Rates of adherence to cervical, breast and colorectal cancer screening are commonly reported for each individual cancer. For example in Canada, participation in cervical, breast, and colorectal screening was 70.2%,[4] 46%,[5] and 16.1% [6] respectively. However, for groups in which screening for more than one cancer is indicated (such as in women older than 50 years of age) participation in more than one cancer screening could also be measured. Women between 50 and 69 years of age, for example, are advised to undergo screening for cervical, breast and colorectal cancers, and those in the 70 to 74 age group should be screened for breast and colorectal cancers. For these, an integrated indicator of participation would go beyond each cancer to quantify women screened for two or all three cancers; unless specifically measured, this cannot be inferred from participation in each of three cancer screenings (Fig 1).

There are multiple barriers to achieve an integrated measure of participation in breast, cervical and colorectal cancers, such as the availability of an electronic infrastructure to support data linkage of personal information across different databases. Ideally, databases of clients’ participation in cancer screening would cover large geographic areas to avoid missing clients who are screened in a different jurisdiction. Finally, confidentiality rules of sharing private information across providers may deter access to the data for the indicator. Despite the challenges, integrated cancer screening has been measured in at least one jurisdiction in Canada; in Ontario in 2013, participation in the three cancer screening programs among women 50–69 years old was 35%.[7]

A performance measure that integrates participation across breast, cervical and/or colorectal cancer screenings can improve the quality of cancer screening programs by allowing refinement and balancing of strategies for different target groups. On the one hand, individuals who are up-to-date with *some* screening may be more open and responsive to receiving additional screenings compared to those who haven't had any screening. For example, women in each section of the Venn diagram (except those in the middle, who are already screened for all cancers they are eligible for) may have different needs, and would therefore benefit from different strategies to increase uptake for one or more screenings they are missing. On the other hand, evidence suggests that screened adults are generally enthusiastic about screening [8], and may thus be possibly over-screened. An integrated measure would allow the pinpointing of populations at risk of harm from unnecessary testing.

The purpose of this systematic review was to examine the literature for the use of performance indicators of adherence across multiple cancer screening (cervical, breast and colorectal) at the population level.

## Methods

**Fig 2:**
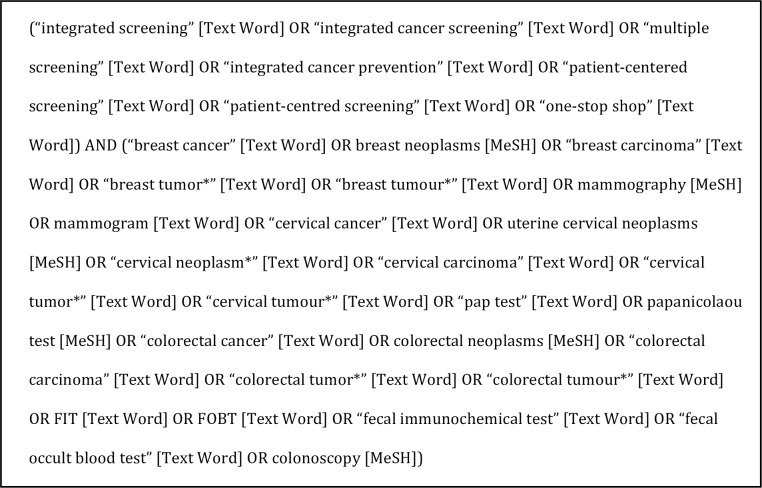
Literature search strategy (applied to all databases).

To identify articles within peer-reviewed literature, a comprehensive search strategy was developed in consultation with a librarian. The key concepts of the search were integrated cancer screening and breast, cervical and colorectal cancer screening. Multiple terms were used to capture literature related to each concept. For integrated cancer screening, search terms included multiple screening, integrated cancer prevention, patient-centred/centered screening, and one-stop shop. For breast, cervical and colorectal cancer screening, the cancer synonyms tumour/tumor, neoplasm and carcinoma were used as well as the following screening tests: mammography, mammogram, papanicolaou test, fecal immunochemical test, fecal occult blood test, colonoscopy, FIT, and FOBT (Fig 2).

Articles were screened at the level of title, then abstract, then full text for relevance by one reviewer. Articles retained at full text were assessed for inclusion based on the following pre-established inclusion criteria outlined in Table 1. Although arbitrary, the rationale for the lower limit of publication date from 2000 onwards was chosen to account for the time frame when CRC screening became more prevalent in Canada [9] and the US [10].

**Table 1:** Inclusion and exclusion criteria for the selection of articles.

	Inclusion criteria	Exclusion criteria
Population	• Women older than 50 years of age	Other age groups
Intervention	• Surveys of clients • Review of administrative information • Medical record review • Data triggered from cancer screening database	• Studies that tested interventions to increase uptake of the three cancer screenings but did not report a combined uptake measure
Outcome	• Proportion of women screened for all three cancers (breast, cervical and colorectal)	• Papers that reported adherence to each cancer screening separately • Other cancer screenings
Other	• Articles published between 2000–2015 • Primary studies or reviews • Written in English • No limits regarding country of study origin or publication	• Any report that did not include an integrated measure of participation in breast, cervical and colorectal cancer screening.

The following seven databases were searched: MEDLINE, EMBASE, EBM Reviews(Cochrane Database of Systematic Reviews, ACP Journal Club, Database of Abstracts of Reviews of Effects, Cochrane Central Register of Controlled Trials, Cochrane Methodology Register, Health Technology Assessment, NHS Economic Evaluation Database), PubMed, PubMed Central, CINAHL, and Nursing Reference Center. Results were combined to identify literature that pertained to cancer screening and breast, cervical and colorectal cancers. A hand search through the reference lists of included articles was done to capture any additional articles.

Several grey literature resources were consulted, including Google, Google Scholar, OpenDOAR (www.opendoar.org), Health Sciences Online [HSO] (www.hso.info), OAISter (http://oaister.worldcat.org), Health Canada (http://www.hc-sc.gc.ca/index-eng.php), Public Health Agency of Canada [PHAC] (http://www.phac-aspc.gc.ca/index-eng.php), and the National Cancer Institute [NCI] (http://www.cancer.gov).

## Results

**Fig 3:**
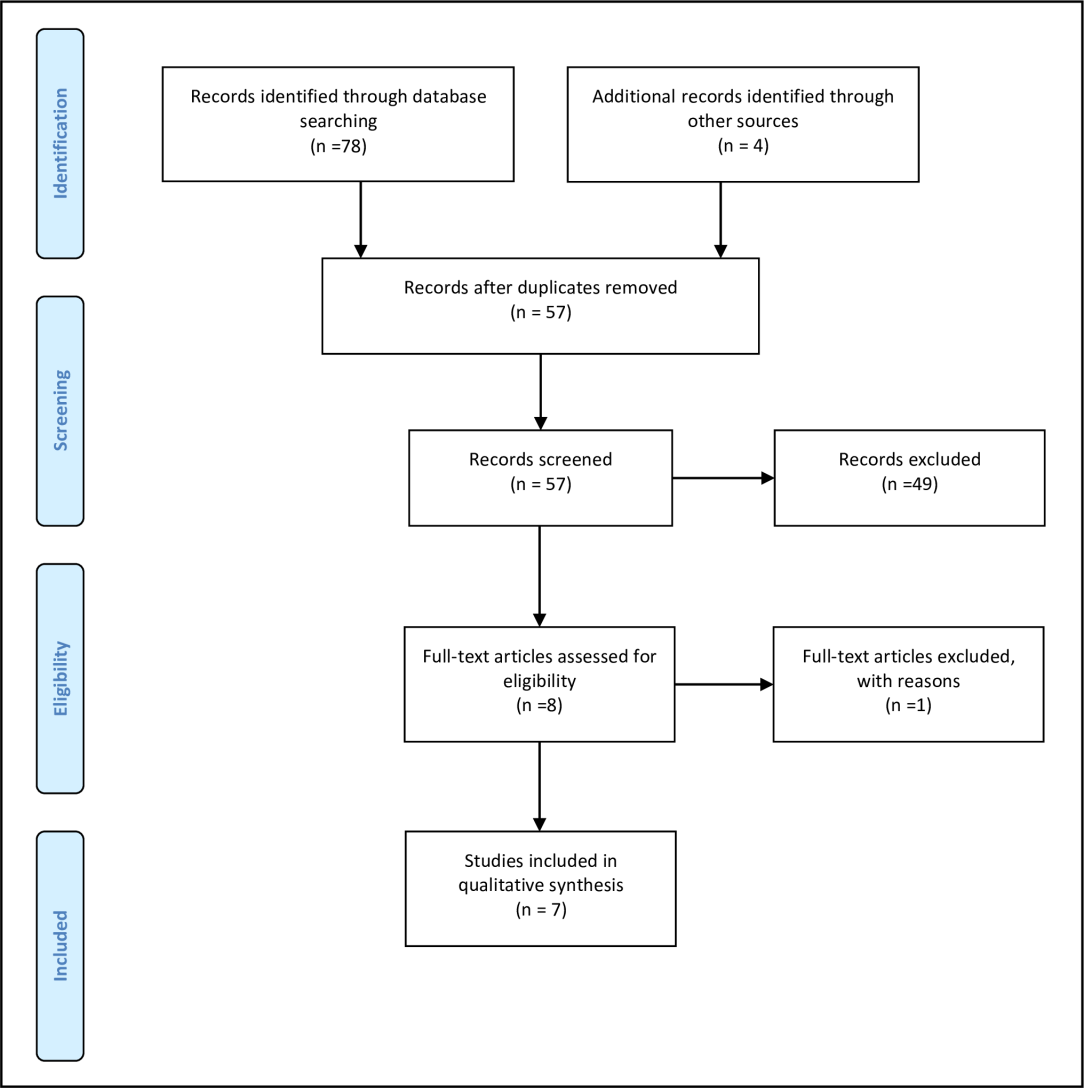
PRISMA Flow Diagram [11] of Integrated Screening: Breast, Cervical, and Colorectal Cancer.

The search strategy is outlined in Fig 2. The strategy identified 78 potential articles, supplemented with an additional five records located through grey literature. Upon removal of duplicates and screening via title/abstract and inclusion/exclusion criteria, eight papers were read in full-text, seven of which are included in this review (Fig 3 and S1 PRISMA Checklist).

Seven studies reported an integrated measure of participation across breast, cervical and colorectal cancer screening, three cross-sectional studies, [12–14] two randomized controlled trials, [15–16] an evaluation of a triple screening pilot program, [17] and a group pre-post intervention study.[18] The indicators of integrated screening for the three cancers were 51.4% in the pilot evaluation [17], and ranged from 16% to over 8% in the three cross sectional studies.[12–14] In the two randomized trials, the tested intervention almost doubled the number of women screened for all three cancers to 43% [16] and 19% [15]. None of the studies included uptake of combinations of 2 specific cancer screenings (such as breast and cervical, but no colorectal). Only, one study [16] reported uptake measures for “two or more cancer screenings” in the intervention and usual care groups, but details about which specific two cancer screenings were not provided. The seven studies included are summarized in Table 2.

**Table 2:** Summary of included studies.

Emmons et al, 2011 [12]	• **Methods:** Cross-sectional descriptive analysis of comprehensive cancer screening practices of 43,000 active primary care patients. Data was extracted from electronic medical records in a large community health center in Massachusetts. Being current with recommended cancer screening tests was based on US preventive service task force and American cancer society clinical guidelines. • **Participants:** Individuals (predominantly low income Latino) eligible for screening who had at least one visit with a primary care provider within the previous 2 years. • **Screening interval and tests:** Being current with recommended cancer screening tests was defined as 2 years for Pap test, and 1 year for mammography. Colorectal cancer screening was defined as colonoscopy within 10 years, or FOBT within 1 year. Three extra months were allowed as a data collection buffer. • **Intervention:** none • **Outcomes:** Only women in the 50–70 year age group were eligible for all three screening tests. The multiple cancer screening prevalence was 16% for the 50–70 age group, 24% for the 71+ age group.
Katz et al, 2015 [13]	• **Methods:** Cross sectional survey (telephone interview) followed by medical record review (MRR) of participants who reported being within recommended American cancer society screening guidelines for breast, cervical and colorectal cancer. • **Participants:** 637 women 51–75 years old from randomly selected households from 12 Appalachian Ohio counties. • **Screening interval and tests:** Being current with recommended cancer screening tests was defined as 3 years for Pap test, and 1 year for mammography. Colorectal cancer screening was defined as colonoscopy within 10 years, FOBT within 1 year, or flexible sigmoidoscopy within 5 years. • **Intervention:** none • **Outcomes:** Screening rates were 36.1% for cervical cancer, 32.1% for breast cancer, and 30.1% for colorectal cancer. Although almost a third of the women self-reported being within recommended screening guidelines for all three tests, only 8.6% had completed all three tests as per MRR.
Billette de Villemeur et al, 2007 [17]	• **Methods:** Evaluation of a pilot triple screening program. Participation in breast, cervical and colorectal cancer screening was extracted from the screening center database at the end of the pilot and covering the period 1990–1996. Participation was also assessed through a telephone opinion survey of a random sample of the population of invited women. • **Participants:** 98,017 women 50–69 years old living in Isère, France. • **Screening interval and tests:** Women were invited every 30 months to participate in breast (mammography) or cervical (Pap test) screening programs, and every two years for pilot CRC (FOBT) screening. • **Intervention:** A pilot combined screening program for breast, cervical and colorectal cancer screening in Isère. A first round of invitation letters were mailed to 87,643 women in 1990, and a second round of letters were sent to 90,382 women two-and-a-half years later. The letter invited women to consult their gynecologist or general practitioner. During the visit the physician performed a Pap test, explained modalities of colorectal cancer screening and prescribed a screening mammography. • **Outcomes:** Among the invited women 39.1% and 41.2% of those who received the first and second invitation respectively attended screening. Of those who attended, 51.4% received all three screening tests, while 27.6% received two and 17.9% received only one screening test. When surveyed, 44% of the invited women reported being current with all three tests.
Allen et al, 2014 [18]	• **Methods:** One group pre/post evaluation design of five evidence-based interventions (EBI) with self-reported compliance with screening guidelines. Definitions of adherence were based on the American Cancer Society. • **Participants:** Seventy-seven women age 18 or over, and members of a Latino Baptist church in Boston, MA. Only 36 (47%) of those who completed the pre-intervention assessment also completed the post-intervention assessment. • **Screening interval and tests:** Compliance with breast and cervical cancer screening were defined as mammogram within 2 years, and Pap test within 3 years respectively. CRC screening was defined as annual FOBT, sigmoidoscopy within 5 years or colonoscopy within 10 years. • **Intervention:** The following five EBIs were conducted over a six-month period: one-to-one education, group education, dissemination of health messages via small media and pastor sermons, behavioral goal-setting, and reducing structural barriers (i.e. provider referrals, mobile health vans, assistance with applications for state-based insurance) • **Outcomes:** Self-reported adherence with screening guidelines between pre- and post- intervention among women who completed the follow-up assessment showed a 24% increase in adherence with breast cancer screening recommendations (n = 13 pre- and n = 18 post-intervention), and an 8% increase in adherence to all recommended screening tests for one’s age (n = 24 pre- and 27 post-intervention). These changes did not reach statistical significance.
Dietrich et al. 2007 [15]	• **Methods:** Randomized trial of 1,316 women identified from the database of a Medicaid managed care organization (MMCO) in New York City. Administrative data was analyzed on intent-to treat regardless of whether successful contact was made, and a subgroup analysis of women who did actually receive the intervention. Definitions of up-to-date status were derived from US Preventive Services Task Force guidelines • **Participants:** Eligibility criteria were women 40–69 years of age, received care at 1 of 6 participating Community Health Centers, had been enrolled with the MMCO for at least 12 months, and were overdue for at least 1 of the targeted cancer screening tests. • **Screening interval and tests:** Up-to-date with breast and cervical cancer screenings, namely a mammography within 2 years, and a Pap test within 3 years respectively. CRC screening pertained to women who had FOBT within 1 year, sigmoidoscopy or double-contrast enema within 5 years, or colonoscopy within 10 years. • **Intervention:** Women in the study were assigned to either a prevention care management intervention (PCM) or a comparison group. Both groups received up to three scripted telephone calls, scheduling assistance for breast cancer screening, a financial incentive for receiving a mammogram, and mailed educational material on breast, cervical and colorectal cancer screening. In addition, women in the PCM group received support to identify and overcome barriers to obtain breast, cervical and colorectal cancer screening. • **Outcomes:** Screening rates for cervical and breast cancer did not differ significantly between study groups, however PCM seemed to impact colorectal screening uptake on an intent-to-treat basis. At follow up in all women aged 50 years or older, women assigned to PCM were almost twice as likely to be up-to-date for all 3 tests (59 of 317, 18.6%) versus their counterparts assigned to the comparison group (33 of 309, 10.7%); this difference was statistically significant.
Dietrich et al. 2006 [16]	• **Methods:** Randomized controlled trial of 11 migrant health centers in New York City. Medical record reviewed for evidence of screening for breast, cervical and/or colorectal cancers followed the US Preventive Services Task Force recommendations. • **Participants:** Women between 50–69 years of age who were overdue for at least one cancer screening as per their medical records, were patients of the center for at least 6 months and had no plans to move within the next 15 months. • **Screening interval and tests:** Mammography and Pap tests that were performed within one year were seen as evidence of breast and cervical cancer screening, respectively. FOBT within the past year, sigmoidoscopy within the past 5 years, or colonoscopy within the past 10 years were seen as evidence of colorectal cancer screening. • **Intervention:** Several reminder phone calls in which case managers provide support, address screening barriers, schedule appointments and arrange transportation. • **Outcomes** Patient medical records reviewed >3 months after the 18 month intervention period. Participation in all three-cancer screenings from baseline increased from 21% to 43%, in the intervention group (n = 696); and from 22 to 30% in the usual care group (n = 694).
Carlos et al. 2004 [14]	• **Methods:** Telephone survey of a representative sample of the population. The aim of the survey was to understand the relationship between cancer screening behaviors to enhance colorectal cancer screening. Adherence to the American Cancer Society guidelines of colorectal cancer screening was the primary outcome and breast and cervical adherence were used as independent predictors of colorectal cancer adherence. • **Participants:** Women 50 years of age and older who participated in the 2000 Behavioral Risk Factors Surveillance Survey, and lived in any of the 5 US states where the colorectal module was administered. • **Screening interval and tests:** Up-to-date for cervical and breast cancer screening were women who reported having a Pap test within 3 years, and a mammography within 1 year respectively. For CRC screening women were considered compliant if they reported having an FOBT within the previous year, or a sigmoidoscopy or colonoscopy within the previous 5 years. • **Intervention:** none • **Outcomes:** A total of 2788 women participated in the survey but only 1300 responded to the colorectal cancer module. Of these, adherence to colorectal, cervical and breast was 24.9%, 57.2% and 78.6% respectively. Only 114 (8.76%) women reported being screened for the tree cancers.

Six of seven studies [12–16, 18] were conducted in the US, and one was from France.[17] Four of the US studies addressed disadvantaged groups including Latino, [12,18] Appalachian, [13] and minority and low income women. [16] Clients’ screening histories were collected from electronic medical records, [12,16] administrative data, [15] self- report, [14,18] or a combination of self-report followed by a review of administrative data. [13,17] Only the French study [17] extracted data from a cancer screening database.

The main purpose of the studies were to evaluate interventions to increase cancer screening behavior, [15,16,18] document inequities in cancer screening, [12,13] report the results of a pilot program, [17] and test whether cervical and breast screening adherence are predictors of compliance with colorectal cancer screening.[14] The largest sample sizes were used in the pilot evaluation (around 100,000 participants) [17] and in one of the cross sectional studies (n = 43,000), while for the remaining five studies, sample sizes ranged from around 77 to 3,000 women.

One of the cross sectional studies [12] noted that adherence to multiple screening dropped as the number of tests that women were eligible for increased. Pap test uptake was 65% among young women for which cervical cancer screening is the only screening recommended, but only 16% of women 50–70 years old (eligible for cervical, breast and colorectal cancer screening) and 24% of women >70 years of age (eligible for colorectal and breast cancer screening) regularly underwent all screening tests they were eligible for. In one cross sectional study, [13] 31.9% of the surveyed women reported being current with all three cancer screening tests but only 8.6% were confirmed up-to-date on the subsequent medical record review. Another survey [14] noted that women who adhered to both breast and cervical cancer screening were more likely to adhere to colorectal cancer compared to women who adhered to cervical cancer screening alone; self-reported participation in the three cancer screenings was 8.8%.

In a pilot program [17] of combined triple screening, about 90% of women living in an administrative district of France were invited to participate. Data from the cancer screening database showed that around 40% of the invited women attended, of whom 55% received the triple screening. Therefore, overall adherence with the three cancer screenings was around 22% of the eligible population.

The two randomized controlled trials were conducted by the same group of researchers.[15,16] They tested a Prevention Care Management (PCM) intervention to increase participation in breast, cervical and colorectal cancer screening among a minority, low-income population of women in New York City. Participation in the first (intensive setting) and second (practical) trials showed higher but modest participation among women in the PCM versus usual care groups.

Finally, Allen et al, 2014 [18] assessed the feasibility, acceptability and impact of a church-based educational program to promote cancer screening among Latinas. There was substantial attrition and a non-significant self-reported increase in compliance with breast cancer screening (n = 5) and all tests recommended for age (n = 3). This latter category did not provide details on women’s age or participation in multiple cancer screening tests.

## Discussion

The purpose of this study was to review the literature for the use of performance indicators of adherence across cervical, breast and colorectal cancer screening. The number of studies identified was very limited, perhaps due to the fact that integrated indicators are used as internal performance evaluation programs and may not get published. However, reports of adherence to single cancer screening abound in the literature. Among studies that reported integrated screening, levels of participation in all three cancers ranged from slightly over 50% to 8%. Overall, the purpose of the studies included in this review were to document inequities among low income groups, [12–13]^,^ and to test interventions to increase uptake of cancer screening among vulnerable women, [15–16, 18] or at the population level [17]. Overall, interventions were successful in increasing adherence to multiple screening except for one study with a small sample size, in which the intervention did not reach statistical significance in increasing screening uptake.[18] As expected, results from the studies that used a combination of a self-report followed by a review of administrative data [13] or cancer screening database [17] confirmed that self-reporting may overestimate participation in multiple cancer screening.

The paucity of literature reporting on integrated cancer screening adherence is curious given that population-based surveys (such as the National Health Interview Survey in the US and the Canadian Community Health Survey)[19–21] having the potential to report integrated measures of participation in cancer screening fail to do so. Instead, they report adherence for each cancer screening individually. Only one study included in this literature review used population-based survey responses and concluded that women who adhere to breast and cervical cancer screening were significantly more likely to adhere to colorectal cancer screening, compared to women who adhered to either cervical or breast cancer screening alone.

Integration of cancer screening services is challenging [22] because tests include differing body sites, target groups and testing intervals that vary depending on age, and comfort of the test provider in administering screening and interpreting test results. As such, we were interested in determining whether this literature review would show that adherence with breast and cervical cancer is more common than any other combination of two cancer screenings, as a result of women obtaining screening at “well women” or healthcare visits dedicated to gynecologic health where providers may not be as familiar with colorectal cancer screening. Unfortunately none of the papers included in this review reported on uptake of cervical and breast, or any other combination of two cancer screenings; only uptake of individual cancer screening or the three combined were included.

In the context of a client-centered delivery approach that aims to deliver services in a way that satisfies clients’ needs, [23] (such as a “one-stop-shop” for cancer screening) integrated measures of adherence seek to address all screenings for which an individual is eligible for [24]. An integrated approach to the provision of cancer screening is highly consistent with a patient-centered orientation to medical care and more fruitful than separate visits for each cancer screening test. [12] Integrated cancer screening delivery requires its own targets for performance indicators [25] that reflect uptake of all cancer screenings that clients are eligible for. These indicators can position cancer screening programs as well as individual providers to better understand the needs of their target population and clients. Different messages and strategies could be used if the goal is to recruit screening naïve women into cancer screening compared to women who already participate in one or more cancer screenings, and those at risk of harms from over-screening.

In summary, this review identifies a gap in the literature around integrated adherence to breast, cervical and colorectal cancer screening, which can provide additional information on the screening needs of the population, and have the potential to add value to process improvement initiatives. Integrated participation complements performance measures of individual programs, with implications that can extend beyond cancer screening to other population-based interventions with specific target groups, such as non-cancer screening (e.g. infant hearing screening) and immunizations. Integrated measures of participation may become more widespread in the future with advances in information technology to support linkages of population level data.”

## Supporting Information

S1 PRISMA Checklist PRISMA 2009 Checklist. (DOC)
